# (*E*)-1-(Pyridin-2-yl)ethanone *O*-acryloyloxime

**DOI:** 10.1107/S1600536808005436

**Published:** 2008-03-05

**Authors:** Mariusz Mojzych, Zbigniew Karczmarzyk, Andrzej Fruziński

**Affiliations:** a Department of Chemistry, University of Podlasie, ul. 3 Maja 54, 08-110 Siedlce, Poland; b Institute of General and Ecological Chemistry, Technical University, ul. Żwirki 36, 90-924 Łódź, Poland

## Abstract

The title compound, C_10_H_10_N_2_O_2_, was synthesized by the reaction of the oxime of 2-acetyl­pyridine and 3-bromo­propanoyl chloride in the presence of triethyl­amine. The mol­ecule adopts a nearly planar chain-extended conformation with the oxime group in a *trans* and the acryloyl group in an *s*-*cis* conformation. This conformation is stabilized by an intra­molecular C—H⋯N hydrogen bond. The screw-related mol­ecules are linked into *C*(9) chains by C—H⋯O hydrogen bonds.

## Related literature

For general background, see: Robertson, (1995 ▶). For the biological activity of oximes, see: Van Helden *et al.* (1996 ▶). For related structures, see: Mojzych *et al.* (2007 ▶). For the graph-set notation, see: Bernstein *et al.* (1995 ▶).
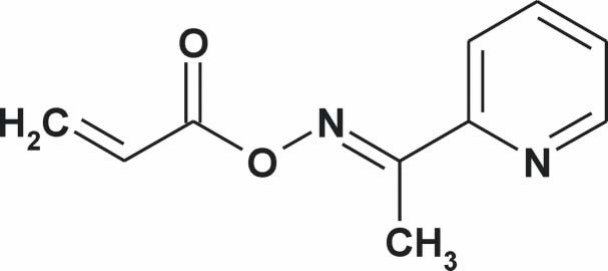


## Experimental

### 

#### Crystal data

C_10_H_10_N_2_O_2_
*M* *_r_* = 190.20
Monoclinic, 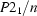

*a* = 7.0240 (5) Å
*b* = 18.4054 (14) Å
*c* = 7.8642 (6) Å
β = 114.043 (1)°
*V* = 928.47 (12) Å^3^
*Z* = 4
Mo *K*α radiation
μ = 0.10 mm^−1^
*T* = 100 (2) K
0.75 × 0.13 × 0.07 mm

#### Data collection

Bruker SMART APEXII CCD diffractometer
Absorption correction: multi-scan (*SADABS*; Sheldrick, 2002 ▶) *T*_min_ = 0.924, *T*_max_ = 0.993
15216 measured reflections
2209 independent reflections
1951 reflections with *I* > 2σ(*I*)
*R*_int_ = 0.035

#### Refinement

*R*[*F* ^2^ > 2σ(*F* ^2^)] = 0.033
*wR*(*F* ^2^) = 0.098
*S* = 1.02
2209 reflections
167 parameters
All H-atom parameters refined
Δρ_max_ = 0.40 e Å^−3^
Δρ_min_ = −0.18 e Å^−3^

### 

Data collection: *SMART* (Bruker, 2003 ▶); cell refinement: *SAINT* (Bruker, 2003 ▶); data reduction: *SAINT*; program(s) used to solve structure: *SIR92* (Altomare *et al.*, 1993 ▶); program(s) used to refine structure: *SHELXL97* (Sheldrick, 2008 ▶); molecular graphics: *ORTEP-3 for Windows* (Farrugia, 1997 ▶); software used to prepare material for publication: *SHELXL97* (Sheldrick, 2008 ▶), *PLATON* (Spek, 2003 ▶) and *WinGX* (Farrugia, 1999 ▶).

## Supplementary Material

Crystal structure: contains datablocks I, global. DOI: 10.1107/S1600536808005436/ci2565sup1.cif

Structure factors: contains datablocks I. DOI: 10.1107/S1600536808005436/ci2565Isup2.hkl

Additional supplementary materials:  crystallographic information; 3D view; checkCIF report

## Figures and Tables

**Table 1:** Hydrogen-bond geometry (Å, °)

*D*—H⋯*A*	*D*—H	H⋯*A*	*D*⋯*A*	*D*—H⋯*A*
C10—H10*B*⋯N1	0.94 (2)	2.438 (14)	2.8596 (14)	107 (1)
C1—H1⋯O2^i^	0.96 (2)	2.588 (15)	3.4581 (14)	150 (1)

Symmetry code: (i) 
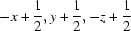
.

**Fig. 1.**
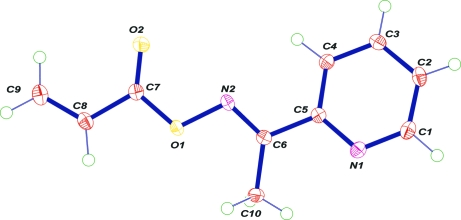
The molecular structure of the title compound, showing the atom-numbering scheme. Displacement ellipsoids are drawn at the 50% probability level.

**Fig. 2.**
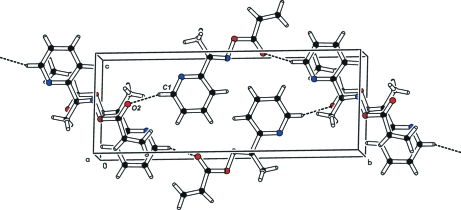
A view of the molecular packing in the title compound. Dashed lines indicate intermolecular hydrogen bonds.

## supplementary crystallographic information

### Comment

Oximes and their derivatives such as *O*-ethers and esters are important intermediates in organic chemistry and are well known in both analytical and coordination chemistry (Robertson, 1995). These compounds are also of interest as biologically active compounds (Van Helden *et al.*, 1996). In this in mind we have decided to synthesize and structurally characterize a set of *O*-acryloyl oximes of 6-membered *aza*-heterocycles as 1,2,4-triazine, pyridine and pyrazine. These compounds were obtained by reaction of appropriate oximes and 3-bromopropanoyl chloride under Staudinger reaction conditions. As a part of our ongoing studies, we report herein the crystal and molecular structure of the title compound.

The geometric parameters (bond lengths, angles and torsion angles) in the title compound are very similar to those observed in a previously reported structure of (*E*)-1-(3-methylsulfanyl-1,2,4-triazin-5-yl)-ethanone *O*-acryloyl oxime (Mojzych *et al.*, 2007). The oxime group is in *trans* and the acryloyl group in s-*cis* conformation with the torsion angles O1—N2—C6—C5 and O2—C7—C8—C9 of 179.39 (7) and 2.73 (16)°, respectively. The molecule as a whole adopts a nearly planar chain-extended conformation (Fig. 1). This conformation is stabilized by an intramolecular C10—H10B···N1 hydrogen bond leading to the formation of a five-membered ring described by the S(5) graph-set symbol (Bernstein *et al.*, 1995).

In the crystal structure, the screw-related molecules are linked to form C(9) chains along the [010] direction by C1—H1···O2 intermolecular hydrogen bonds (Fig. 2).

### Experimental

To a solution of 2-acetylpyridine (204 mg, 1.5 mmol) and triethylamine (454 mg, 4.5 mmol) in dry CH_2_Cl_2_ (5 ml) at 233 K was added 3-bromopropionyl chloride (1.5 mmol) in CH_2_Cl_2_ (2 ml) dropwise. The reaction mixture was allowed to warm to room temperature and was stirred for 12 h. It was then washed with water (2 × 10 ml), saturated aqueous sodium bicarbonate (3 × 10 ml), brine (1 × 10 ml) and dried over MgSO_4_. Removal of the solvent yielded the crude product which was then purified by column chromatography on silica gel using CH_2_Cl_2_-hexane mixture (2:1) as eluent to afford the title compound as a colourless solid. Yield: 216 mg (76%) and m.p. 338 K. Single crystals suitable for X-ray diffraction analysis were grown by slow evaporation of an ethanol solution. ^1^H NMR (CDCl_3_) δ: 2.55 (s, 3H), 5.99–6.02 (d, 1H, J = 10.5 Hz), 6.29–6.38 (dd, 1H, J = 10.5 Hz), 6.59–6.65 (d, 1H, J = 17.4 Hz), 7.34–7.38 (t, 1H, J = 6.6 Hz), 7.72–7.78 (t, 1H, J = 8.1 Hz), 8.12 - 8.15 (d, 1H, J = 8.1 Hz), 8.65–8.67 (d, 1H, J = 6.6 Hz). ^13^C NMR (CDCl_3_) δ: 13.11, 122.28, 125.27, 126.72, 132.59, 136.78, 149.34, 152.89, 163.71, 164.28. HR—MS (m/*z*) for C_10_H_11_N_2_O_2_: 191.0822 [*M*^+^+H]; calcd. 191.0821.

### Refinement

All H atoms were located in a difference Fourier map and were refined isotropically [C—H = 0.929 (15)–0.989 (15) Å].

### Crystal data

**Table:**

C_10_H_10_N_2_O_2_	*F*_000_ = 400
*M**_r_* = 190.20	*D*_x_ = 1.361 Mg m^−^^3^
Monoclinic, *P*2_1_/*n*	Melting point: 338 K
Hall symbol: -P 2yn	Mo *K*α radiation λ = 0.71073 Å
*a* = 7.0240 (5) Å	Cell parameters from 166 reflections
*b* = 18.4054 (14) Å	θ = 3.8–28.0º
*c* = 7.8642 (6) Å	µ = 0.10 mm^−^^1^
β = 114.043 (1)º	*T* = 100 (2) K
*V* = 928.47 (12) Å^3^	Prism, colourless
*Z* = 4	0.75 × 0.13 × 0.07 mm

### Data collection

**Table:**

Bruker SMART APEXII CCD diffractometer	1951 reflections with *I* > 2σ(*I*)
Radiation source: fine-focus sealed tube	*R*_int_ = 0.035
*T* = 100(2) K	θ_max_ = 28.4º
ω scans	θ_min_ = 2.2º
Absorption correction: multi-scan(SADABS; Sheldrick, 2002)	*h* = −9→9
*T*_min_ = 0.924, *T*_max_ = 0.993	*k* = −24→24
15216 measured reflections	*l* = −10→10
2209 independent reflections	

### Refinement

**Table:**

Refinement on *F*^2^	Secondary atom site location: difference Fourier map
Least-squares matrix: full	Hydrogen site location: difference Fourier map
*R*[*F*^2^ > 2σ(*F*^2^)] = 0.033	All H-atom parameters refined
*wR*(*F*^2^) = 0.098	*w* = 1/[σ^2^(*F*_o_^2^) + (0.0531*P*)^2^ + 0.3208*P*] where *P* = (*F*_o_^2^ + 2*F*_c_^2^)/3
*S* = 1.03	(Δ/σ)_max_ = 0.001
2209 reflections	Δρ_max_ = 0.40 e Å^−^^3^
167 parameters	Δρ_min_ = −0.18 e Å^−^^3^
Primary atom site location: structure-invariant direct methods	Extinction correction: none

### Special details

**Table:**

Geometry. All e.s.d.'s (except the e.s.d. in the dihedral angle between two l.s. planes) are estimated using the full covariance matrix. The cell e.s.d.'s are taken into account individually in the estimation of e.s.d.'s in distances, angles and torsion angles; correlations between e.s.d.'s in cell parameters are only used when they are defined by crystal symmetry. An approximate (isotropic) treatment of cell e.s.d.'s is used for estimating e.s.d.'s involving l.s. planes.
Refinement. Refinement of *F*^2^ against ALL reflections. The weighted *R*-factor *wR* and goodness of fit *S* are based on *F*^2^, conventional *R*-factors *R* are based on *F*, with *F* set to zero for negative *F*^2^. The threshold expression of *F*^2^ > σ(*F*^2^) is used only for calculating *R*-factors(gt) *etc*. and is not relevant to the choice of reflections for refinement. *R*-factors based on *F*^2^ are statistically about twice as large as those based on *F*, and *R*- factors based on ALL data will be even larger.

### Fractional atomic coordinates and isotropic or equivalent isotropic displacement parameters (Å^2^)

**Table:**

	*x*	*y*	*z*	*U*_iso_*/*U*_eq_	
O1	0.29133 (11)	−0.01660 (4)	0.63817 (9)	0.01600 (18)	
O2	0.12879 (12)	−0.12207 (4)	0.50393 (10)	0.02091 (19)	
N1	0.27586 (13)	0.17974 (5)	0.26737 (12)	0.0168 (2)	
N2	0.23265 (12)	0.01032 (5)	0.45236 (11)	0.0149 (2)	
C1	0.22032 (15)	0.21070 (6)	0.09936 (15)	0.0189 (2)	
C2	0.14439 (15)	0.17215 (6)	−0.06672 (15)	0.0189 (2)	
C3	0.13150 (15)	0.09699 (6)	−0.06045 (14)	0.0176 (2)	
C4	0.19186 (15)	0.06379 (6)	0.11195 (14)	0.0157 (2)	
C5	0.25793 (14)	0.10712 (5)	0.27155 (14)	0.0139 (2)	
C6	0.30844 (14)	0.07431 (5)	0.45895 (13)	0.0137 (2)	
C7	0.22368 (15)	−0.08629 (5)	0.63995 (14)	0.0148 (2)	
C8	0.28945 (16)	−0.10972 (6)	0.83633 (15)	0.0184 (2)	
C9	0.25144 (17)	−0.17670 (6)	0.87484 (16)	0.0212 (2)	
C10	0.43601 (17)	0.11625 (6)	0.63073 (14)	0.0179 (2)	
H1	0.234 (2)	0.2626 (8)	0.0980 (19)	0.023 (3)*	
H2	0.106 (2)	0.1958 (8)	−0.180 (2)	0.026 (3)*	
H3	0.081 (2)	0.0693 (8)	−0.173 (2)	0.027 (3)*	
H4	0.185 (2)	0.0111 (7)	0.1226 (18)	0.021 (3)*	
H8	0.363 (2)	−0.0726 (8)	0.931 (2)	0.030 (4)*	
H9A	0.293 (2)	−0.1938 (7)	1.002 (2)	0.025 (3)*	
H9B	0.180 (2)	−0.2100 (7)	0.776 (2)	0.025 (3)*	
H10A	0.389 (2)	0.1079 (8)	0.726 (2)	0.038 (4)*	
H10B	0.429 (2)	0.1661 (9)	0.605 (2)	0.039 (4)*	
H10C	0.581 (3)	0.1023 (8)	0.673 (2)	0.039 (4)*	

### Atomic displacement parameters (Å^2^)

**Table:**

	*U*^11^	*U*^22^	*U*^33^	*U*^12^	*U*^13^	*U*^23^
O1	0.0177 (4)	0.0178 (4)	0.0120 (3)	−0.0008 (3)	0.0056 (3)	0.0015 (3)
O2	0.0281 (4)	0.0172 (4)	0.0168 (4)	−0.0010 (3)	0.0085 (3)	−0.0012 (3)
N1	0.0150 (4)	0.0154 (4)	0.0200 (4)	0.0006 (3)	0.0072 (3)	0.0004 (3)
N2	0.0158 (4)	0.0173 (4)	0.0119 (4)	0.0022 (3)	0.0059 (3)	0.0022 (3)
C1	0.0168 (5)	0.0153 (5)	0.0244 (5)	0.0010 (4)	0.0081 (4)	0.0033 (4)
C2	0.0154 (5)	0.0227 (5)	0.0181 (5)	0.0023 (4)	0.0064 (4)	0.0067 (4)
C3	0.0152 (5)	0.0220 (5)	0.0154 (5)	−0.0014 (4)	0.0060 (4)	−0.0011 (4)
C4	0.0147 (4)	0.0154 (5)	0.0176 (5)	0.0000 (3)	0.0072 (4)	0.0004 (4)
C5	0.0104 (4)	0.0162 (5)	0.0155 (5)	0.0013 (3)	0.0058 (3)	0.0005 (4)
C6	0.0115 (4)	0.0160 (5)	0.0149 (5)	0.0024 (3)	0.0067 (3)	−0.0007 (4)
C7	0.0131 (4)	0.0163 (5)	0.0169 (5)	0.0031 (3)	0.0080 (4)	0.0013 (4)
C8	0.0167 (5)	0.0237 (5)	0.0156 (5)	0.0008 (4)	0.0073 (4)	0.0013 (4)
C9	0.0212 (5)	0.0241 (6)	0.0208 (5)	0.0039 (4)	0.0110 (4)	0.0051 (4)
C10	0.0205 (5)	0.0181 (5)	0.0154 (5)	−0.0027 (4)	0.0077 (4)	−0.0025 (4)

### Geometric parameters (Å, °)

**Table:**

O1—C7	1.3698 (12)	C4—C5	1.3971 (14)
O1—N2	1.4352 (10)	C4—H4	0.976 (13)
O2—C7	1.2005 (13)	C5—C6	1.4954 (13)
N1—C1	1.3426 (14)	C6—C10	1.4957 (13)
N1—C5	1.3442 (13)	C7—C8	1.4843 (14)
N2—C6	1.2849 (13)	C8—C9	1.3224 (15)
C1—C2	1.3879 (15)	C8—H8	0.989 (15)
C1—H1	0.962 (14)	C9—H9A	0.972 (15)
C2—C3	1.3884 (15)	C9—H9B	0.954 (14)
C2—H2	0.929 (15)	C10—H10A	0.947 (17)
C3—C4	1.3868 (14)	C10—H10B	0.937 (16)
C3—H3	0.954 (15)	C10—H10C	0.969 (16)
			
C7—O1—N2	112.13 (7)	N2—C6—C5	113.73 (8)
C1—N1—C5	116.97 (9)	N2—C6—C10	126.54 (9)
C6—N2—O1	109.45 (8)	C5—C6—C10	119.74 (8)
N1—C1—C2	123.73 (10)	O2—C7—O1	125.00 (9)
N1—C1—H1	116.3 (8)	O2—C7—C8	126.30 (9)
C2—C1—H1	120.0 (8)	O1—C7—C8	108.69 (8)
C3—C2—C1	118.79 (9)	C9—C8—C7	120.19 (10)
C3—C2—H2	120.2 (9)	C9—C8—H8	124.2 (9)
C1—C2—H2	121.0 (9)	C7—C8—H8	115.5 (9)
C4—C3—C2	118.38 (9)	C8—C9—H9A	122.2 (8)
C4—C3—H3	121.3 (8)	C8—C9—H9B	120.1 (8)
C2—C3—H3	120.3 (8)	H9A—C9—H9B	117.7 (11)
C3—C4—C5	118.96 (9)	C6—C10—H10A	111.0 (10)
C3—C4—H4	121.0 (8)	C6—C10—H10B	110.3 (10)
C5—C4—H4	120.0 (8)	H10A—C10—H10B	109.1 (13)
N1—C5—C4	123.07 (9)	C6—C10—H10C	109.4 (9)
N1—C5—C6	116.01 (8)	H10A—C10—H10C	109.9 (13)
C4—C5—C6	120.90 (9)	H10B—C10—H10C	107.0 (13)
			
C7—O1—N2—C6	−176.92 (7)	O1—N2—C6—C10	−0.77 (13)
C5—N1—C1—C2	0.84 (14)	N1—C5—C6—N2	160.49 (8)
N1—C1—C2—C3	−2.47 (15)	C4—C5—C6—N2	−18.13 (12)
C1—C2—C3—C4	1.12 (14)	N1—C5—C6—C10	−19.37 (12)
C2—C3—C4—C5	1.61 (14)	C4—C5—C6—C10	162.02 (9)
C1—N1—C5—C4	2.13 (14)	N2—O1—C7—O2	0.98 (13)
C1—N1—C5—C6	−176.46 (8)	N2—O1—C7—C8	−179.69 (7)
C3—C4—C5—N1	−3.38 (14)	O2—C7—C8—C9	2.73 (16)
C3—C4—C5—C6	175.14 (8)	O1—C7—C8—C9	−176.58 (9)
O1—N2—C6—C5	179.39 (7)		

### Hydrogen-bond geometry (Å, °)

**Table:**

*D*—H···*A*	*D*—H	H···*A*	*D*···*A*	*D*—H···*A*
C10—H10B···N1	0.94 (2)	2.438 (14)	2.8596 (14)	107 (1)
C1—H1···O2^i^	0.96 (2)	2.588 (15)	3.4581 (14)	150 (1)

Symmetry codes: (i) −*x*+1/2, *y*+1/2, −*z*+1/2.
